# *In Vivo* 3-D Dose Verification Using PET/CT Images After Carbon-Ion Radiation Therapy

**DOI:** 10.3389/fonc.2021.621394

**Published:** 2021-03-15

**Authors:** Lining Sun, Weigang Hu, Songtao Lai, Leijun Shi, Junchao Chen

**Affiliations:** ^1^Department of Radiation Oncology, Fudan University Shanghai Cancer Center, Shanghai, China; ^2^Department of Oncology, Shanghai Medical College, Fudan University, Shanghai, China; ^3^Department of Nuclear Medicine, Shanghai Proton and Heavy Ion Center, Shanghai, China

**Keywords:** carbon ion, radiation therapy, positron emission tomography, standard uptake value, dose verification

## Abstract

**Objective:**

To investigate the usefulness of positron emission tomography (PET) images obtained after carbon-ion irradiation for dose verification in carbon-ion radiotherapy.

**Methods and Materials:**

An anthropomorphic head phantom was used in this study. Three cubes with volumes of 1, 4, and 10 ml were contoured as targets in the phantom CT through a treatment planning system. Treatment plans with six prescriptions from 2.5 to 10 Gy (2.5, 3, 5, 6, 8, and 10 Gy effective dose) were designed and delivered by 90° fixed carbon-ion beams, respectively. After irradiation of the phantom, a PET/CT scan was performed to fuse the treatment-planning CT image with the PET/CT image. The relationship between target volume and the standard uptake value (SUV) in PET/CT was evaluated for corresponding plan prescription. The MIM Maestro software was used for the image fusion and data analysis.

**Results:**

SUV in the target had an approximate linear relationship with the effective dose. The same effective dose could generate a roughly equal SUV for different target volumes (*p* < 0.05).

**Conclusions:**

It is feasible to verify the actual 3-D dose distribution of carbon-ion radiotherapy by the approach in this study.

## Introduction

Radiation therapy is one of the primary methods in cancer treatment. To improve treatment outcomes, high accuracy of radiotherapy technology is emphasized. For ion-beam radiotherapy, particle therapy–positron emission tomography (PT-PET) is becoming popular for treatment verification. Although there are a number of radiation dose-verification methods, none can be used directly in 3-D patient dose verification. PT-PET is currently the only clinically applied method for *in vivo* verification. This study proposes an approach using PET/CT images and standard uptake value (SUV) after carbon-ion radiotherapy for *in vivo* 3-D dose verification. During carbon-ion irradiation, ^11^C (half-life T_1/2_ ≈ 20 min) is formed in nuclear interactions between the ions and the tissue mainly within the range of the carbon-ion Bragg peak. Distribution and radioactivity of this positron emitter can be detected *via* PET/CT shortly after therapy, which indicates the distribution of dose deposited by carbon ions. The carbon-ion dose distribution should correspond to the prescription in the treatment plan. To achieve a goal of 3-D dose verification, the relationship between the dose prescribed in the target volume and SUV values on corresponding images from a PET/CT scan was analyzed quantitatively.

## Materials and Methods

### Materials and Experiment Device

**Figure 1:**
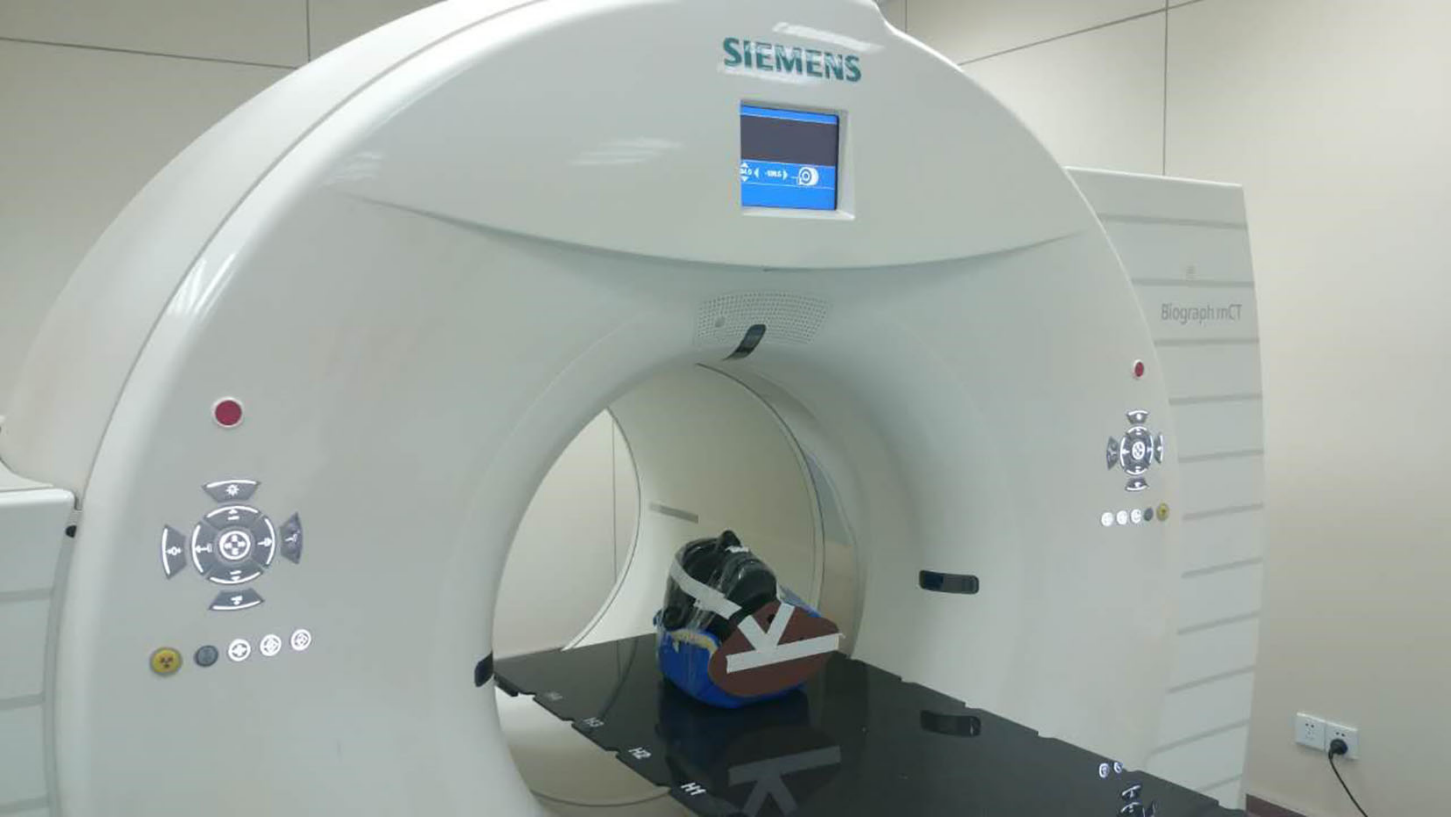
Diagram of carbon-ion virtual target irradiation experimental plan.

A Rando head phantom (The Phantom Laboratory, Salem, NY) was used in this study, and a SIEMENS Definition AS 64 CT simulator was used to acquire the planning image of the phantom. All the phantom plans were created by the Syngo VIA Version 12 particle Treatment Planning System. A SIEMENS Biograph mCT system was used for PET/CT scan (**Figure 1**). The phantom was scanned with a PET/CT scanning scope of 60.5 cm (transverse) by 21.6 cm (axial) with a 3-D maximum expected ordered subset (3-D-OSEM) image reconstruction algorithm, point dispersion function, and line time algorithm.

### Principle and Method

During the carbon-ion irradiation, positron emitters are formed on the beam path *via* nuclear fragmentation reactions, which can be acquired with a PET/CT scanner. A relatively low *β*^+^ activity of 1.6k Bq cm^−3^ is formed per gray of therapeutic dose in the tissue under the carbon-ion irradiation.

For heavier ions (such as ^16^O), the fragmentation reaction can happen on both the incident particles (projectile fragmentation) and target nuclei (target fragmentation) (1–3). Then target dose distribution calculated by the treatment planning system (TPS) and the positron activity distribution of the target region in actual irradiation are compared to find out the relationship between them to decide whether it is feasible to do the PET-based target dose verification for carbon-ion radiotherapy.

### CT Immobilization and Irradiation Planning

The Alfa-cradle foam was used to fix the Rando head phantom. The markers placed on the surface of the phantom defined the original zero point of the CT scanning axis. In addition, the accuracy of the irradiation field size was known in this study. There were no other possible motions of the phantom.

The clinical head tumor CT scanning protocol was applied for this study (axial supine position, 512*512 pixel dimension, slice thickness 1.5 mm, tube voltage 120 kVp and 300 mAs current). After scanning, all CT images were imported to the TPS for the virtual target contouring. Some cubes (virtual CTV) were created to represent different clinical target volumes for chordoma patients in the TPS. Volumes of the cubes were 10, 4, and 1 ml. A single 90° fixed carbon-ion beam was delivered for each virtual CTV and effective fraction doses were from 2.5 to 10 Gy (2.5, 3, 5, 6, 8, and 10 Gy), respectively.

Eighteen groups of different target volume and dose combinations (6*3) were created to generate virtual treatment plans. Clinical treatment parameters (full width at half maximum (FWHM) = 3.0 mm, ripple filter level = 3.0 mm, and dose calculation grid = 2.0 mm) were applied in this study to do the planning optimization. In the single-beam optimization mode, the optimum solution was chosen as the virtual treatment plan after at least 50 iterations.

A dose-volume histogram (DVH) was created for each treatment plan. In the DVH, a 100% prescription dose completely covered more than 95% of the virtual target volume for nine groups of tests.

### Phantom Study

The Rando head phantom was used to simulate a clinical patient treatment workflow on the IONTRIS facility. In total, 18 beam plans that had various target volumes and different target doses were created. Before each plan delivery, the accuracy of the phantom setup was verified by the integrated orthogonal X-ray image system. The tolerance of setup deviation was ±1 mm in each direction. The time of plan delivery was recorded to calculate decay time of radioactive elements (**Table 1**). All carbon-ion beams were raster scanning pencil beams with an energy range from 174.5 to 248.6 MeV.

**Table 1:** Time list of beam-on time and phantom transit time of every delivery (unit: min).

Single Fraction Dose (Gy)	Target volume	Real “beam on” time (T_irr_)	Phantom transit time (Δt)	PET/CTScanning time (T_PET_)
2.5 Gy	CTV-1 ml	1:20	5:15	30:00:00
3 Gy	CTV-1 ml	1:25	5:20	30:00:00
5 Gy	CTV-1 ml	1:36	4:40	30:00:00
6 Gy	CTV-1 ml	1:38	4:58	30:00:00
8 Gy	CTV-1 ml	1:58	4:56	30:00:00
10 Gy	CTV-1 ml	2:10	4:52	30:00:00
2.5 Gy	CTV-4 ml	1:43	5:22	30:00:00
3 Gy	CTV-4 ml	1:50	5:30	30:00:00
5 Gy	CTV-4 ml	1:55	5:19	30:00:00
6 Gy	CTV-4 ml	2:01	5:22	30:00:00
8 Gy	CTV-4 ml	2:03	5:16	30:00:00
10 Gy	CTV-4 ml	2:10	5:12	30:00:00
2.5 Gy	CTV-10 ml	2:33	5:23	30:00:00
3 Gy	CTV-10 ml	1:50	5:30	30:00:00
5 Gy	CTV-10 ml	2:41	5:18	30:00:00
6 Gy	CTV-10 ml	2:01	5:22	30:00:00
8 Gy	CTV-10 ml	2:50	5:02	30:00:00
10 Gy	CTV-10 ml	3:00	5:42	30:00:00

### PET/CT Scan After Carbon-Ion Beam Irradiation

**Figure 2:**
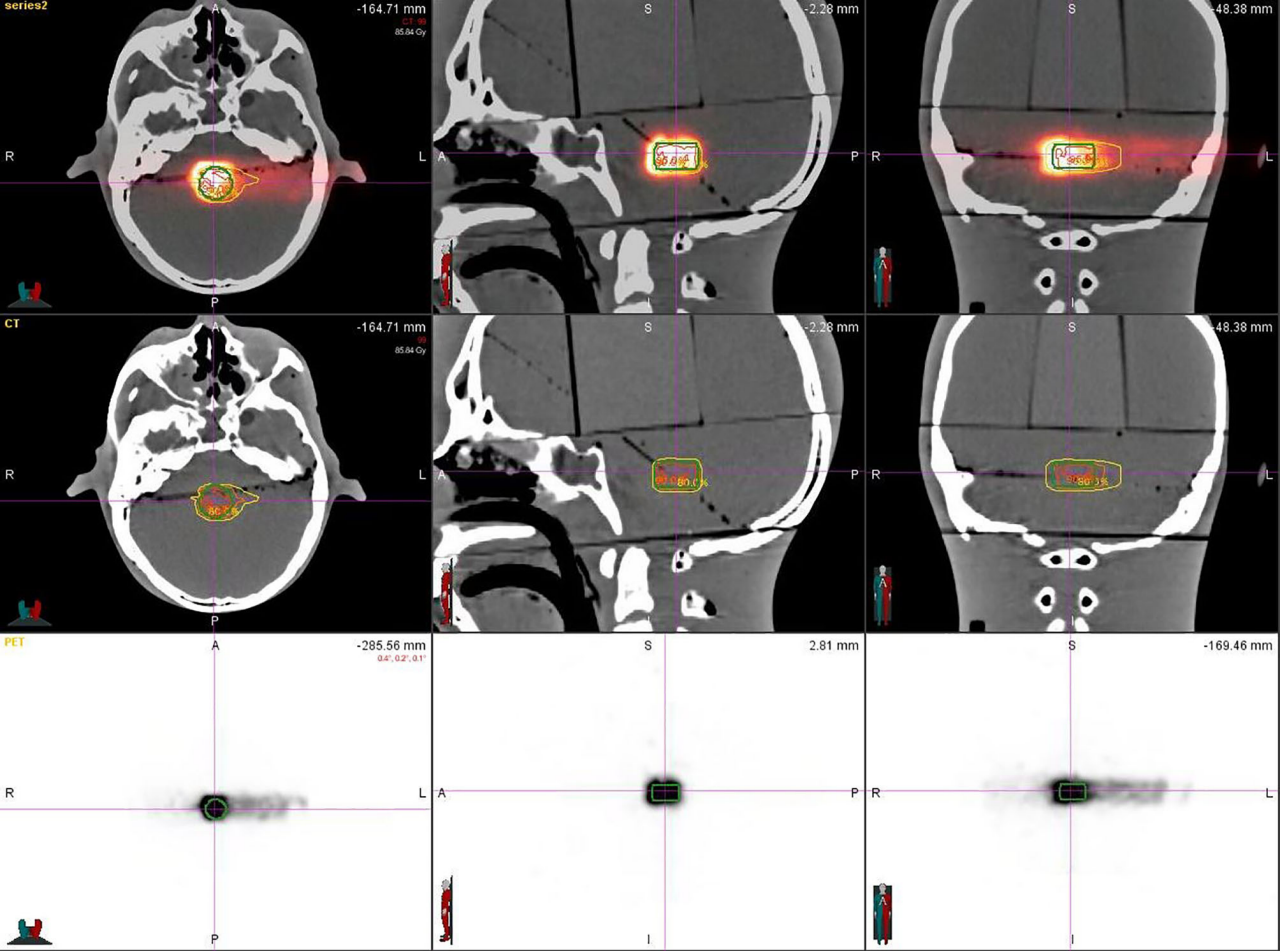
Rando head phantom PET/CT scanning.

After plan delivery, the head phantom was quickly transferred to the PET/CT room within 6 min, which was much shorter than the half-life of the ^11^C (t_1/2_ = 20.38 min), to get PET/CT images with high signal-to-noise ratio (SNR). After the scanning setup, we referred to the routine diagnosis SUV value of the PET/CT image obtained by the 18 FDG radiopharmaceutical using the parameters of the pretest: the virtual body weight is 50 Kg, the virtual radioactive drug injection, the decay time parameter is fine-tuned according to each delivery, and every substudy total scanning time is 30 min. That means, when the scanning is finished, the signal, including a half-life time of the activated substance (^11^C) produced by the carbon-ion irradiation in the phantom, was fully collected. Eighteen sets of PET/CT verification images were acquired (**Figure 2**).

### Processing of Image Registration

**Figure 3:**
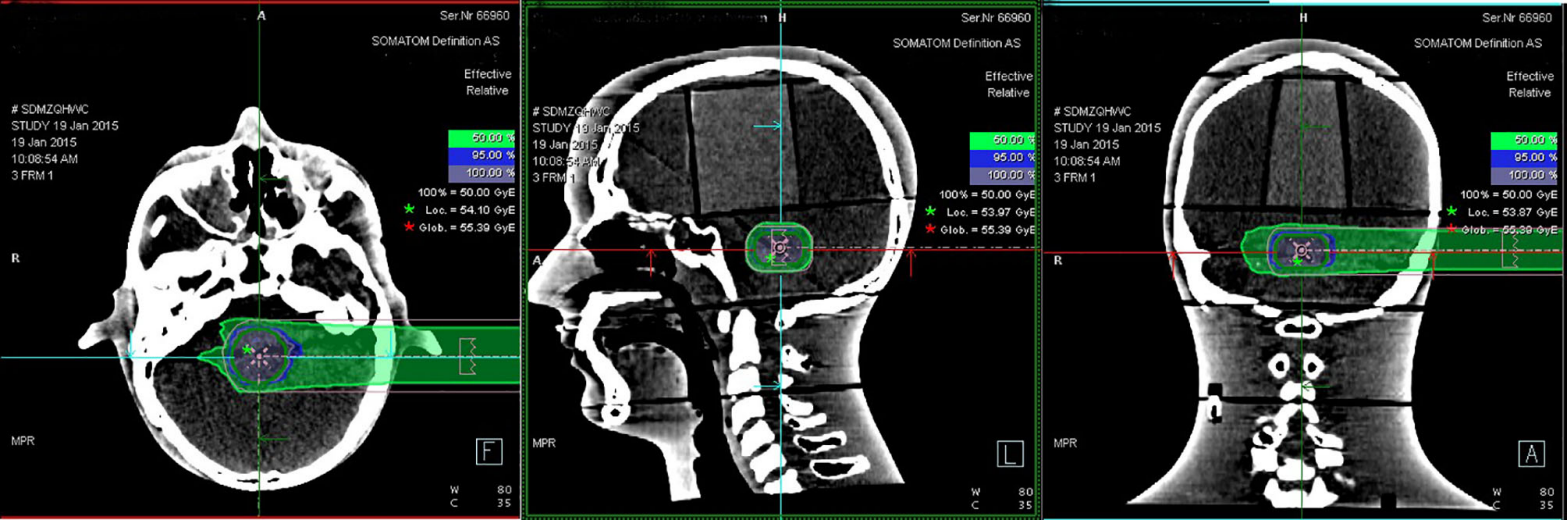
Example of rigid registration fusion for planned CT and PET/CT image.

Eighteen groups of DICOM studies were imported to the MIM Maestro software version 6.5.9 as the reference data for image registration, including CT images, virtual clinical target contours, and the relative effective plan doses. Eighteen sets of PET/CT verification images were also imported. To ensure the accuracy of image registration, we used a rigid registration workflow. After automatic registration by MIM Maestro, slight manual adjustment was done (**Figure 3**).

These experimental data were processed by a linear fitting method after obtaining them for each different volume and dose group. The correlation between these data was also analyzed.

The data statistics function module of the MIM software was used to calculate SUV for each virtual CTV to find out the maximum, minimum, mean, and total SUV of the corresponding target volume. To compare the differences in the same set obtained with the same relative dose to different volume targets, the SPSS statistical analysis software Version 22 was used to perform rigid alignment on the fusion images of each group. The maximum, minimum, and mean SUV data were also processed by this software. The difference of SUV data between variant target volumes was analyzed by independent sample *t* test.

## Results

### Experimental Research Data Analysis

The virtual CTV was registered to the corresponding structure of the PET/CT image for statistical analysis. We get the following results listed in **Table 2**:

**Table 2:** SUV of each target volume under different carbon-ion irradiation doses.

Single Fraction Dose	Target Volume	Max SUV	Mean SUV	Min SUV	Total SUV
2.5 Gy	CTV-1ml	5.91	2.67	1.5	80.32
3 Gy	CTV-1ml	7.18	3.24	1.82	97.59
5 Gy	CTV-1ml	16.92	10.17	2.69	307.38
6 Gy	CTV-1ml	17.23	10.71	4.37	371.93
8 Gy	CTV-1ml	31.51	22.6	9.86	684.42
10 Gy	CTV-1ml	35.53	23.39	9.42	706.97
2.5Gy	CTV-4ml	6.49	3.54	1.56	426.27
3 Gy	CTV-4ml	7.72	4.21	1.86	507.26
5Gy	CTV-4ml	17.08	11	2.87	1315.56
6 Gy	CTV-4ml	17.76	11.37	4.27	1369.61
8Gy	CTV-4ml	36.38	24.19	11.03	2812.43
10 Gy	CTV-4ml	38.43	26.40	12.34	3223.12
2.5Gy	CTV-10ml	6.83	3.45	1.31	1069.95
3 Gy	CTV-10ml	7.99	4.04	1.53	1251.84
5Gy	CTV-10ml	16.09	9.8	2.55	3010.65
6 Gy	CTV-10ml	18.38	10.90	3.53	3379.97
8Gy	CTV-10ml	35.88	20.73	9.23	6438.01
10 Gy	CTV-10ml	37.01	22.05	10.25	6773.96

**Figure 4:**
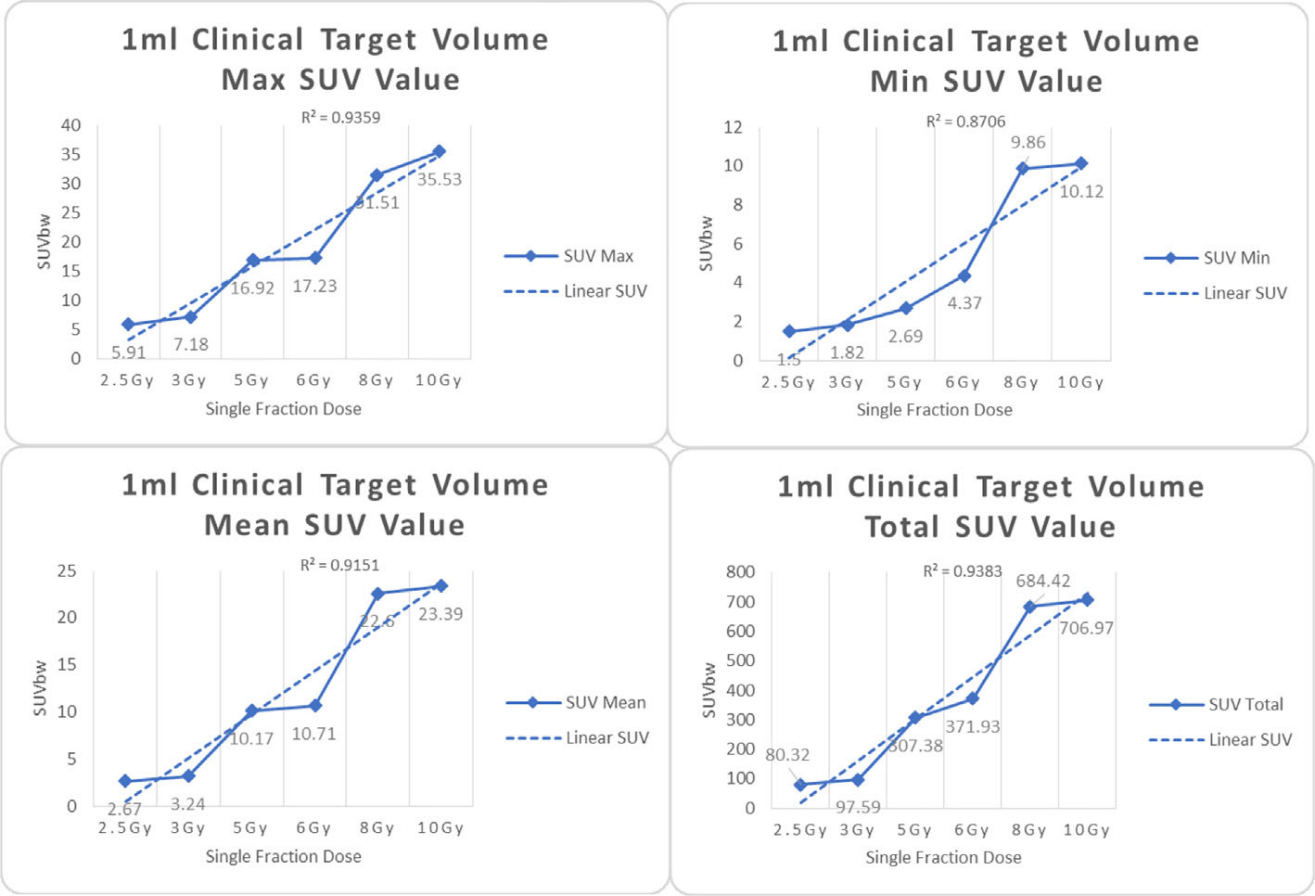
Maximum, minimum, average, and total SUV of 1 ml clinical volume for different target doses.

**Figure 5:**
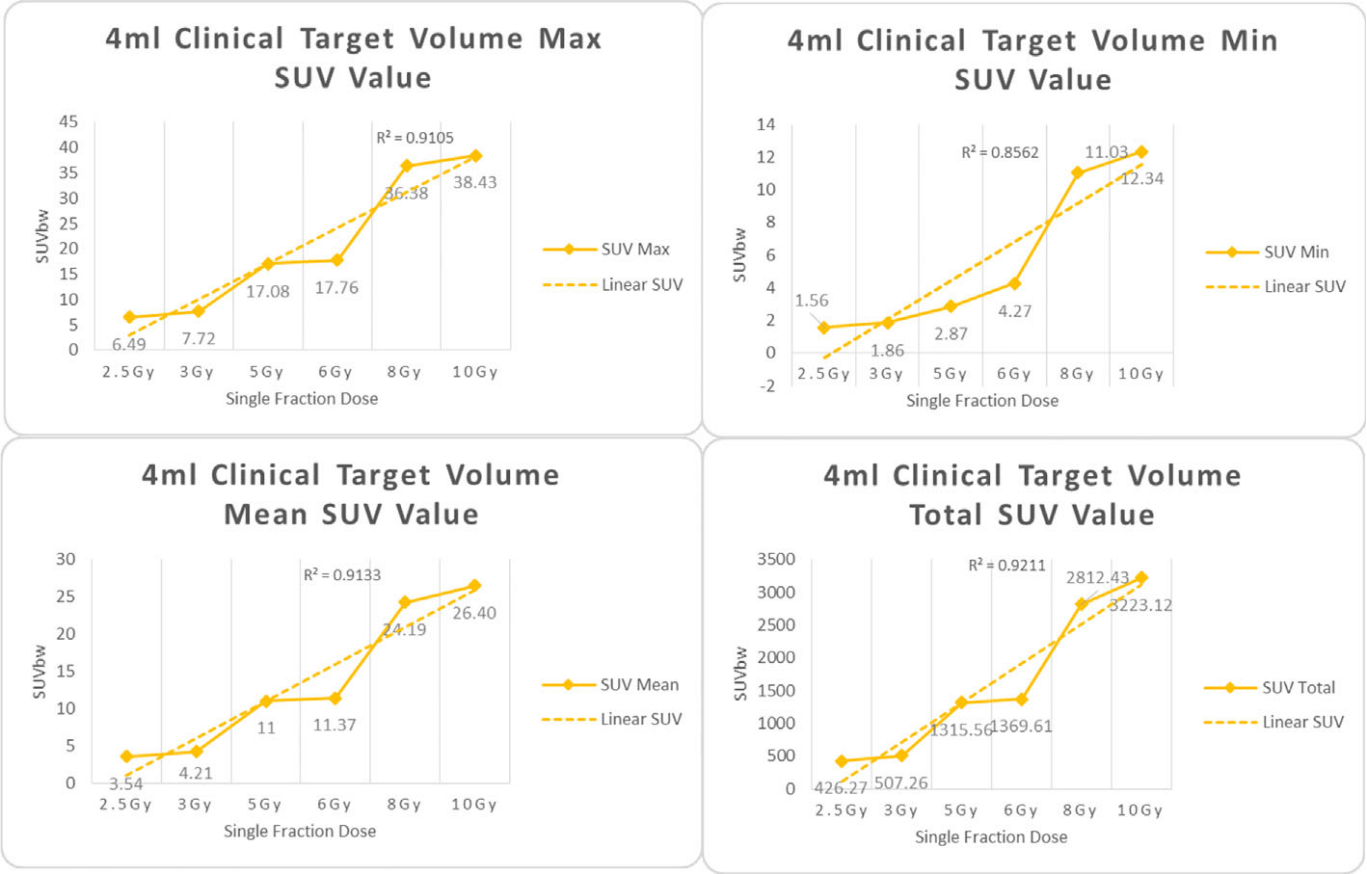
Maximum, minimum, average, and overall SUV of 4 ml clinical volume for different target doses.

**Figure 6:**
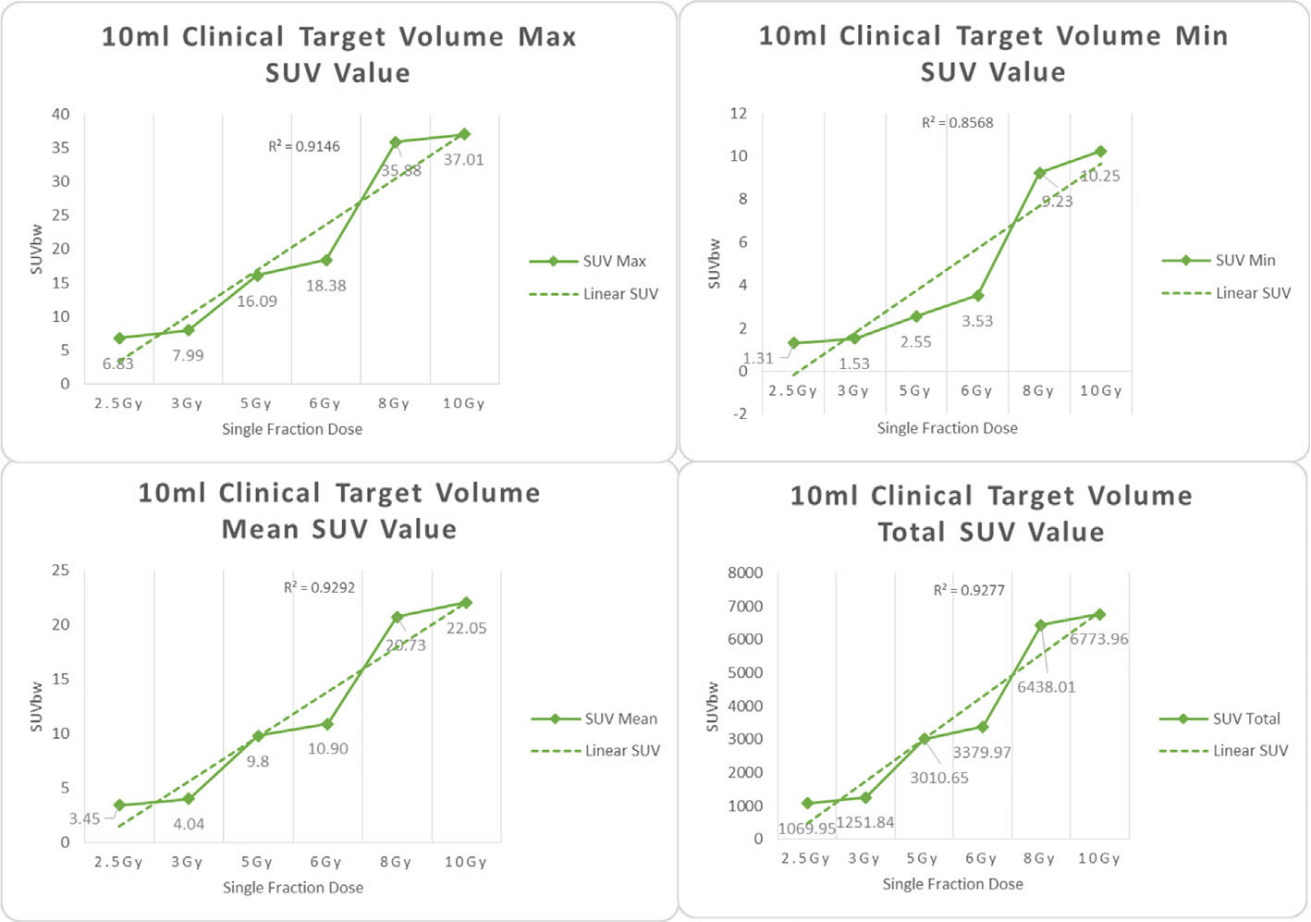
Maximum, minimum, average, and overall SUV of 10 ml clinical volume for different target doses.

In the experimental data, the maximum, minimum, average, and total SUV values of three groups of different target sites were measured after different doses of carbon-ion irradiation, the results were showed on the **Figures 4**–**6**. The SPSS statistical analysis software version 22 was used to perform rigid alignment on the fusion images of each group and the *R*^2^ value of each group was calculated.

For *R*^2^ comparison, the results are as follows:

The six sets of histograms show SUV of single fraction doses from 10 to 2.5 Gy for different target volumes. It can be found that the maximum, minimum, or mean SUV for the different target volumes with same single fraction dose do not have statistically significant differences (*P* > 0.05).

**Figure 7:**
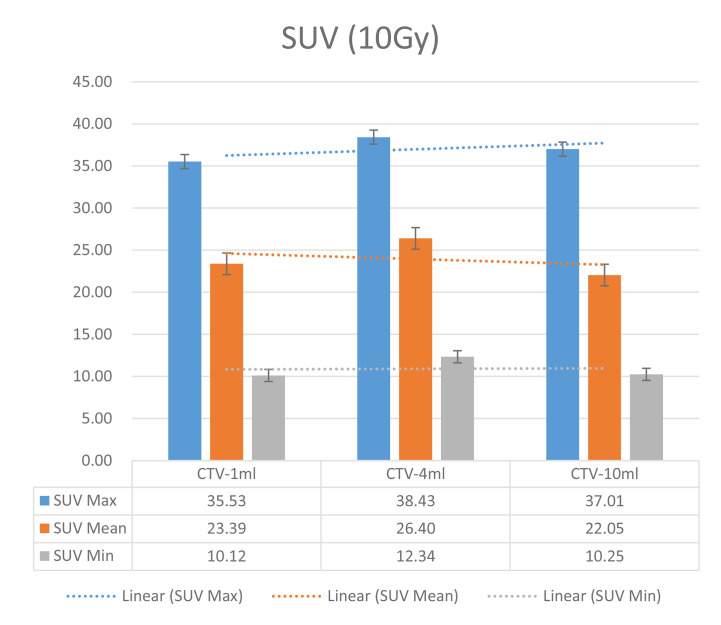
SUV comparison of different target volumes after 10 Gy single fraction dose irradiation.

**Figure 8:**
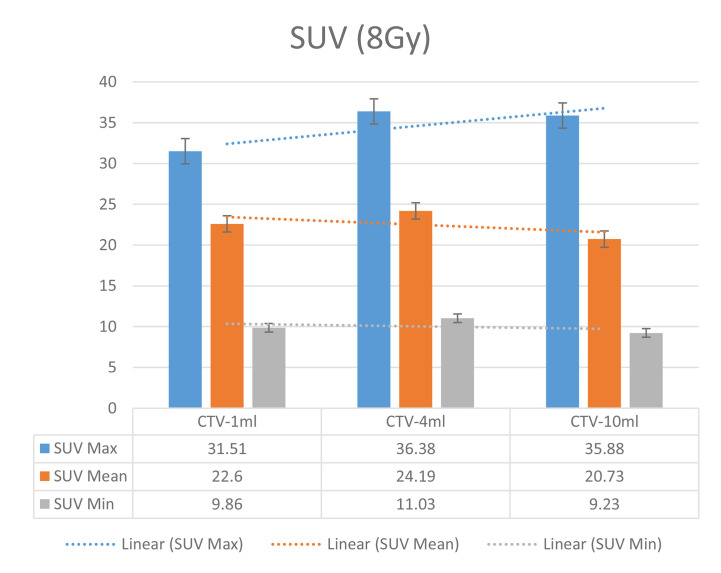
SUV comparison of different target volumes after 8 Gy single fraction dose irradiation.

**Figure 9:**
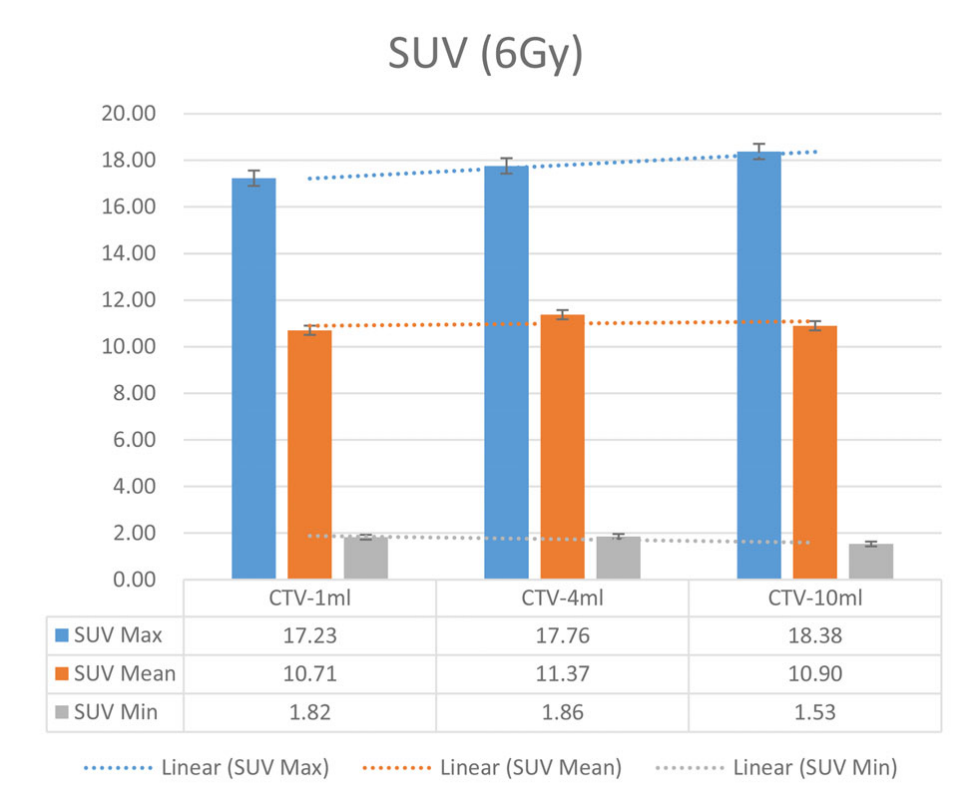
SUV comparison of different target volumes after 6 Gy single fraction dose irradiation.

**Figure 10:**
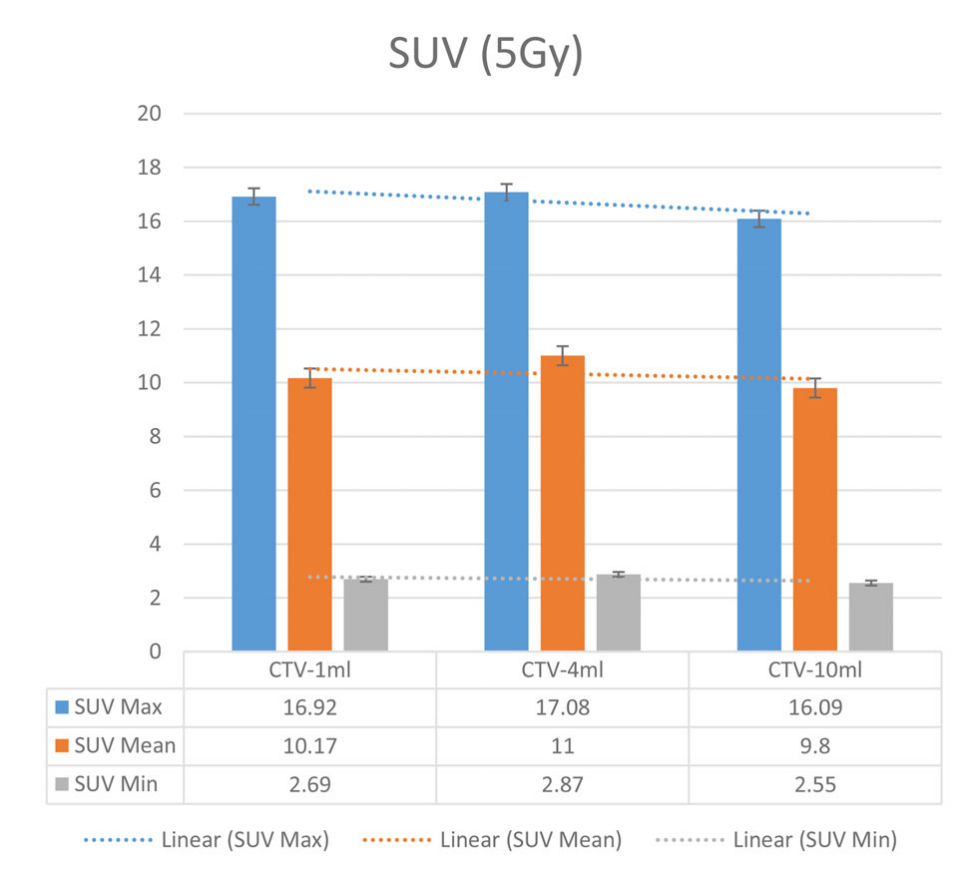
SUV comparison of different target volumes after 5 Gy single fraction dose irradiation.

**Figure 11:**
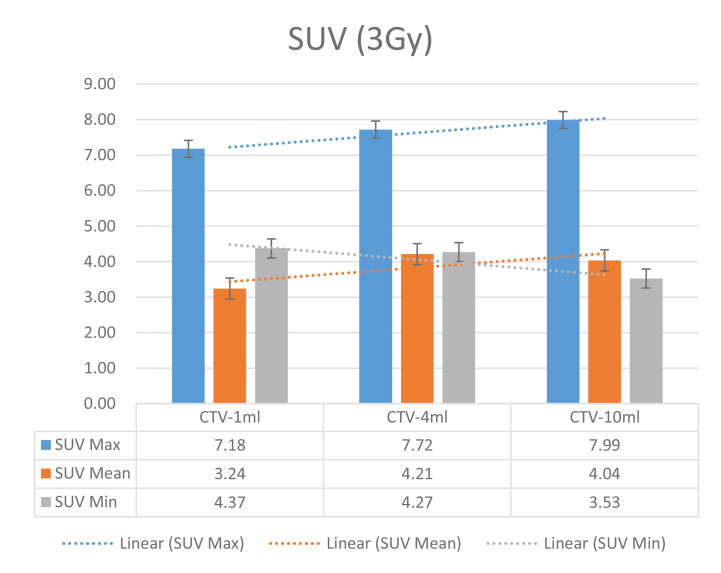
SUV comparison of different target volumes after 3 Gy single fraction dose irradiation.

**Figure 12:**
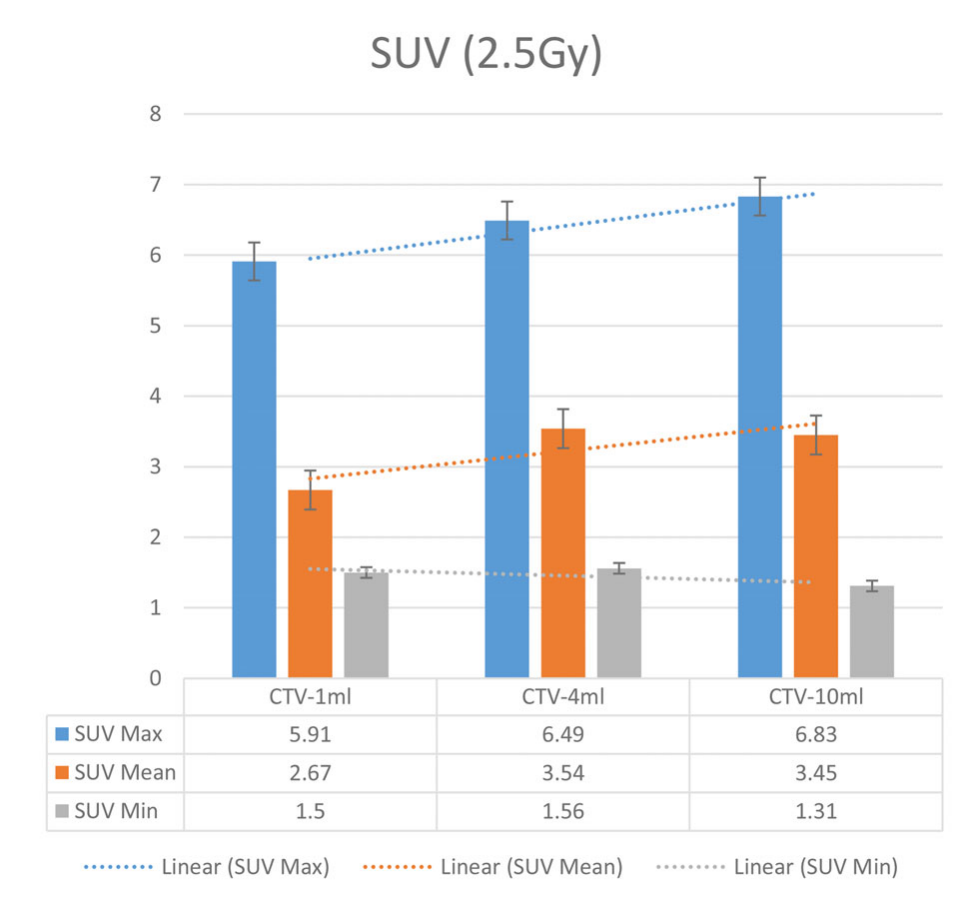
SUV comparison of different target volumes after 2.5 Gy single fraction dose irradiation.

From **Figures 7**–**12** and **Table 3**, we can see that, for different delivered doses, *R*^2^ values were approximately equal to 1 for maximum, minimum, and average SUV within the same target volume. This means, for various doses in different target volumes, the ion-induced SUV can have an interdeducible linear relationship with the target volumes. The SUV on PET/CT image could be quantitatively used for dose verification.

**Table 3:** SUV *R*^2^ values for different target volumes.

Target Volume	SUV Max *R*^2^	SUV Mean *R*^2^	SUV Min *R*^2^	SUV Total *R*^2^
CTV-1 ml	0.9359	0.9151	0.8706	0.9383
CTV-4 ml	0.9105	0.9133	0.8562	0.9211
CTV-10 ml	0.9146	0.9292	0.8568	0.9277

From **Table 3** and these six histograms for different volume target area carbon-ion irradiation, corresponding to different irradiation doses, the same volume target area shows the maximum, minimum, and average SUV numerical fit *R*^2^ values similar to 1, that is, different from the irradiation dose for each group of different target volumes of radiation, the target volume generated by the SUV value between the existence of a deductible linear relationship, corresponds to the same volume of the target area due to carbon ions. The gamma photon dose value generated by the beam irradiation induction can be quantified as the SUV value exhibited by the volume in the PET/CT scan image and can be taken from the target SUV in the verification image under certain conditions Dose value can be extended to the use of the target SUV of the accuracy of the dose to verify.

On the other hand, to compare the differences in the same set obtained with the same relative dose to different volume targets, the maximum, minimum, and mean SUV data were also processed by this software. The difference of SUV data between variant target volumes was analyzed by independent sample *t* test.

It can be explicitly seen from **Tables 4****–****9** that, for the same dose irradiated, SUV in virtual CTV with different volumes were not significantly different (*P* > 0.05). The maximum, minimum, and mean SUV values presented on different target volumes were very close to each other.

**Table 4:** Comparison of SUV values for different target volumes that received the same irradiation dose of 10 Gy.

Irradiation dose of virtual target	CTV-1 ml vs CTV-4 ml	CTV-1 ml vs CTV-10 ml	CTV-4 ml vs CTV-10 ml
10 Gy	*P* = 0.787	*P* = 0.873	*P* = 0.644

**Table 5:** Comparison of SUV values for different target volumes that received same irradiation dose of 8 Gy.

Irradiation dose of virtual target	CTV-1 ml vs CTV-4 ml	CTV-1 ml vs CTV-10 ml	CTV-4 ml vs CTV-10 ml
8 Gy	*P* = 0.755	*P* = 0.975	*P* = 0.754

**Table 6:** Comparison of SUV values for different target volumes that received the same irradiation dose 6 of Gy.

Irradiation dose of virtual target	CTV-1 ml vs CTV-4 ml	CTV-1 ml vs CTV-10 ml	CTV-4 ml vs CTV-10 ml
6 Gy	*P* = 0.748	*P* = 0.964	*P* = 0.832

**Table 7:** Comparison of SUV values for different target volumes that received the same irradiation dose 5 Gy.

Irradiation dose of virtual target	CTV-1 ml vs CTV-4 ml	CTV-1 ml vs CTV-10 ml	CTV-4 ml vs CTV-10 ml
5 Gy	*P* = 0.863	*P* = 0.958	*P* = 0.819

**Table 8:** Comparison of SUV values for different target volumes that received the same irradiation dose of 3 Gy.

Irradiation dose of virtual target	CTV-1 ml vs CTV-4 ml	CTV-1 ml vs CTV-10 ml	CTV-4 ml vs CTV-10 ml
3 Gy	*P* = 0.315	*P* = 0.476	*P* = 0.971

**Table 9:** Comparison of SUV values for different target volumes that received the same irradiation dose of 2.5 Gy.

Irradiation dose of virtual target	CTV-1 ml vs CTV-4 ml	CTV-1 ml vs CTV-10 ml	CTV-1 ml vs CTV-10 ml
2.5 Gy	*P* = 0.443	*P* = 0.489	*P* = 0.993

## Discussion

In the PET/CT scan process, the facility control system could use a specialized series of algorithms to correct the persistent decay data of the radioactive substance (^11^C, etc.) in the image reconstruction and acquirement to ensure that the image data processing procedure is correct within the time of one half-life of the radioactive isotope (4–7).

However, if the PET/CT scan start time is much later or more than one half-life of the radioactive isotope after radiotherapy delivery, the SNR of the positron signal would become much lower, and the background noise of the obtained image data would increase heavily. This leads to a degradation of usefulness of the image. Radionuclides formed during carbon-ion irradiation are very unstable and continue to β^+^ decay. Even if the energy of the β^+^ released by ^11^C isotope decay is only 1.0 MeV, this factor should be taken into account if the high image quality is required. In addition, it is necessary to consider the distance of the path of the positrons before annihilation, which would impact the resolution of the PET image.

Compared with the phantom, different tissues in the human body could change the range of SUV on PET/CT images. When the carbon-ion beam is delivered to the human body, the elemental composition of the various tissues on the beam path affect the number of positrons. For example, when the beam goes through the body cavity or low-density part of lung tissue, the corresponding amount of positrons is reduced, which can affect the measured SUV of these relevant regions.

Cyclic metabolism of organisms would also change the distribution of positrons in the body (8, 9). In this study, dose verification experiments on PET/CT images were performed on the Rando phantom without any blood circulation or various tissue motions. However, in living organisms, if the time interval of carbon-ion radiotherapy and PET/CT image scanning is long enough, the nuclide in the irradiated tissue will not only decay by the physical properties, but also be transported to other parts to participate in biological tissue metabolic activity (10–12). This process includes a number of possible blood or tissue fluids circulation, biological tissues within a variety of molecules associated with carbon-ion irradiation generated by a variety of radioactive isotope capture, microcirculation flow, and so on (13–15). Metabolism in the biological tissue also affects the distribution accuracy of these β^+^ positrons, changes the PET/CT imaging quality, and reduces the accuracy of target dose verification. The biological washout must be corrected in real patients because it can affect the activity distribution significantly. For an accurate simulation, it is important to consider the biological washout of β^+^ emitters due to vital functions. Mathematical expressions for washout have mainly been determined by using radioactive beams of ^10^C and ^11^C ions, both β^+^ emitters, to enhance the counting statistics in the irradiated area (16–18). Still, the question of how the choice of autoactivating or nonautoactivating projectile influences the washout coefficients has been unsolved (19). Therefore, if the target dose verification based on the PET/CT image is carried out in the organisms, the possible impact of precision caused by the metabolic process of β^+^ positrons in organisms must be noted.

## Conclusion

Accurate dose verification is an important prerequisite for the safe and effective implementation of radiation therapy. PET/CT after carbon-ion radiotherapy for clinical dose verification has been demonstrated to be a feasible method. Due to the limited experimental conditions, there is some further work needed to be carried out. More data should be collected in the future to find the exact relationship between the positron distribution and prescribed dose distribution.

## Data Availability Statement

The original contributions presented in the study are included in the article/supplementary material. Further inquiries can be directed to the corresponding author.

## Author Contributions

LSun contributed the central idea, analyzed most of the data, and wrote the initial draft of the paper. The remaining authors contributed to refining the ideas, carrying out additional analyses, and finalizing this paper. All authors contributed to the article and approved the submitted version.

## Conflict of Interest

The authors declare that the research was conducted in the absence of any commercial or financial relationships that could be construed as a potential conflict of interest.

## Abbreviations

PET, Positron Emission Tomography; SUV, Standard Uptake Value; TPS, Treatment Planning System; GyE,Gray Effective; 3D-OSEM, three-dimensional maximum expected ordered subset; FWHM, Full Width at Half Maximum; CTV, Clinical Target Volume; SNR, Signal to Noise Ratio; DVH, Dose Volume Histogram.
